# Kihito prevents corticosterone-induced brain dysfunctions in mice

**DOI:** 10.1016/j.jtcme.2021.05.002

**Published:** 2021-05-15

**Authors:** Ryota Araki, Hayato Tachioka, Ayami Kita, Hironori Fujiwara, Kazufumi Toume, Kinzo Matsumoto, Takeshi Yabe

**Affiliations:** a Laboratory of Functional Biomolecules and Chemical Pharmacology, Faculty of Pharmaceutical Sciences, Setsunan University, 45-1 Nagaotoge-cho, Hirakata, Osaka, 573-0101, Japan; b Division of Medicinal Pharmacology, Institute of Natural Medicine, University of Toyama, 2630 Sugitani, Toyama, 930-0194, Japan; c Division of Pharmacognosy, Institute of Natural Medicine, University of Toyama, 2630 Sugitani, Toyama, 930-0194, Japan

**Keywords:** Corticosterone, Depression, Kihito, Spatial memory, Stress

## Abstract

Kihito (KIT; Gui Pi Tang) is a traditional herbal medicine that is used for treatment of neuropsychiatric disorders such as depression, anxiety, neurosis and insomnia in China and Japan. Recently, it has also been shown that KIT improves cognitive dysfunction in patients with Alzheimer's disease. In this study, to investigate the mechanisms underlying the effects of KIT on stress-induced brain dysfunctions such as a depressed state and memory impairment, we examined whether KIT prevents behavioral and neurophysiological abnormalities in mice treated chronically with corticosterone (CORT). CORT (40 mg/kg/day, s.c.) and KIT (1000 mg/kg/day, p.o.) were given to 7-week-old male ddY mice for 14 days. Twenty-four hours after the last treatment, depression-like behavior in the forced swim test, spatial memory in the Barnes maze test, cell survival and the number of new-born immature neurons, dendritic spine density and expression levels of mRNA for neurotrophic factors were analyzed. Depression-like behavior and spatial memory impairment were observed in CORT-treated mice without KIT treatment. Hippocampal cell survival, the number of hippocampal new-born immature neurons, hippocampal and accumbal dendritic spine density and mRNA levels for neurotrophic factors such as glial cell line-derived neurotrophic factor (GDNF) were decreased in CORT-treated mice without KIT treatment. KIT prevented CORT-induced depression-like behavior, spatial memory impairment, and decreases in hippocampal cell survival, the number of hippocampal new-born immature neurons, accumbal dendritic spine density and GDNF mRNA. KIT may ameliorate stress-induced brain dysfunctions via prevention of adverse effects of CORT on cell survival, new-born immature neurons, spine density and neurotrophic factors.

## Graphical abstract

**Figure:**
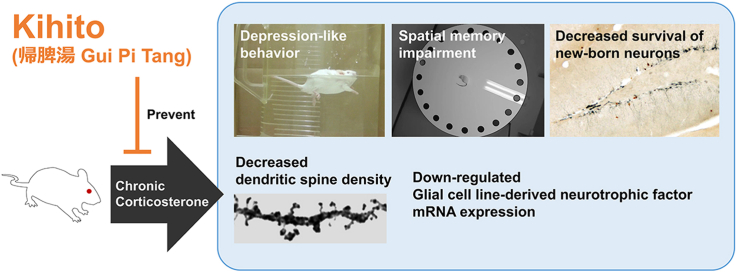

## 1 Introduction

Stress is a common contemporary problem that is difficult to avoid completely. A stress response is a biological reaction to any condition that disrupts the body. One of the major physiological responses to stress is activation of the hypothalamus-pituitary-adrenal (HPA) axis, and consequent synthesis and secretion of glucocorticoids from the adrenal cortex. Glucocorticoids affect numerous physiological processes, including metabolism, immune function, emotion and cognition.^1^^,^^2^ The stress response itself is an integral mechanism for maintenance of homeostasis; however, chronic stress may induce hypersecretion of glucocorticoids, which can cause serious health problems, including mental disorders.^1^

In mice, chronic treatment with corticosterone (CORT), the primary rodent glucocorticoid, induces depression-like behaviors and spatial memory impairments.^3^, ^4^, ^5^ In the brain, hippocampal cell survival, the number of hippocampal new-born immature neurons, hippocampal and accumbal dendritic spine density and mRNA levels for neurotrophic factors are decreased in mice treated chronically with CORT.^4^ Decreases in cell survival and the number of new-born immature neurons reduces neurogenesis, the process through which neural stem and progenitor cells generate new neurons. Adult hippocampal neurogenesis are required for normal behavioral responses to stress or antidepressants^6^^,^^7^ and for spatial reference memory.^8^ Dendritic spines are small protrusions that emerge on the surface of neuronal dendrites, express glutamate receptors, and play a crucial role in neurotransmission. Alterations in dendritic spine density and morphology in the hippocampus and nucleus accumbens are thought to be associated with depression-like behavior, learning and memory in rodents.^9^, ^10^, ^11^ Neurotrophic factors are biomolecules that support proliferation, survival, migration and differentiation of neurons, and also regulate and dendritic spine maturation.^12^, ^13^, ^14^ Therefore, mice treated chronically with CORT are used as an animal model for chronic stress that causes behavioral and neurophysiological abnormalities.^15^

Kihito (KIT; Gui Pi Tang) is a traditional herbal medicine in China and Japan, which is composed of 12 dried medicinal herbs: Astragali Radix (huang qi; dried root of *Astragalus membranaceus* Bunge), Zizyphi Semen (suan zao ren; dried seed of *Zizyphus jujuba* Mill. var. spinosa), Ginseng Radix (ren shen; dried root of *Panax Ginseng* C.A. Meyer), Atractylodis Rhizoma (bai zhu; dried rhizome *Atractylodes japonica* Koidzumi ex Kitamura), Poria (fu ling; dried sclerotium of *Poria cocos* Wolf), Longan Arillus (long yan rou; dried aril of *Euphoria longana* Lam), Polygalae Radix (yuan zhi; dried root of *Polugala tenuifolia*. Willd.), Zizyphi Fructus (da zao; dried fruit of *Zizyphus jujube* Mill. var. inermis Rehd.), Angelicae Radix (dang gui; dried root of *Angelica acutiloba* Kitagawa), Glycyrrhizae Radix (gan cao; dried root of *Glycyrrhiza uralensis* Fisch), Saussureae Radix (mu xiang; dried root of *Saussurea lappa* Clarke) and Zingiberis Rhizoma (sheng jiang; dried rhizome of *Zingiber officinale* Roscoe). KIT is used clinically for a depressed state, anxiety, neurosis, insomnia or anemia in patients with a delicate constitution and a poor complexion. Clinical studies have shown that KIT is effective for Alzheimer-type dementia,^16^^,^^17^ and in preclinical studies, KIT improved amyloid β-induced memory dysfunctions and neuronal atrophy.^18^ However, the mechanisms underlying the ameliorative effects of KIT on stress-induced brain dysfunctions are unclear.

We have previously found that an aqueous extract of Polygalae Radix, a component of KIT, prevents depression-like behavior, decreased dendritic spine density and decreased mRNA levels for glial cell line-derived neurotrophic factor (GDNF) in the hippocampus and nucleus accumbens of mice treated chronically with CORT.^4^ These findings suggest that Polygalae Radix ameliorates a stress-induced depressed state through increasing dendritic spine density and GDNF expression in the hippocampus and nucleus accumbens. This may help with understanding the mechanisms through which Kampo medicines containing Polygalae Radix have effects on brain dysfunctions.

In this study, to investigate the mechanisms of KIT amelioration of stress-induced brain dysfunctions, we examined the effects of KIT on CORT-induced depression-like behavior, spatial memory impairment, and decreases in hippocampal cell survival, the number of hippocampal new-born immature neurons, hippocampal and accumbal dendritic spine density and mRNA for neurotrophic factors.

## 2 Materials and methods

### 2.1 Animals

Experimental procedures concerning the use of animals were approved by the committee for Ethical Use of Experimental Animals at Setsunan University and conducted according to the Guide for the Care and Use of Laboratory Animals (National Research Council, 1996). Every effort was made to minimize suffering and to reduce the number of animals used. Seven-week-old male ddY mice were obtained from Shimizu Laboratory Supplies (Kyoto, Japan) and housed in cages (24 × 17 × 12 cm^3^) in groups of 5 mice under controlled environmental conditions (23 ± 1 °C; 12:12-h light-dark cycle, humidity of 55%, food and water ad libitum).

### 2.2 Drug preparation, treatment and experimental schedule

The component herbs of KIT are shown in Table 1. The Kampo formula was decocted with 10 vol of distilled water for 45 min at 90 °C, followed by immediate filtration of the extract through filter paper in vacuo. The filtrate was spray dried and the yield of KIT extract was approximately 24.3% of the herbal mixture, based on dry weight. The chemical profiling of KIT extract using liquid chromatography-mass spectrometry technique was performed as described in the supplementary information. Chromatographic fingerprinting of KIT is shown in the Wakan-Yaku DataBase system (https://dentomed.toyama-wakan.net/en/information_on_experimental_kampo_extracts/kihito%20extract-2017-KM/EXP009002, Institute of Natural Medicine, University of Toyama). A voucher specimen (No. 20000008) has been deposited at the Institute of Natural Medicine, University of Toyama.

**Table 1:** List of component herbs of kihito.

Component herbs	Amount	Manufacturer
Astragali Radix	3.0 g	Tochimoto Tenkaido Co Ltd., Osaka, Japan
Zizyphi Semen	3.0 g	Tochimoto Tenkaido Co Ltd., Osaka, Japan
Ginseng Radix	3.0 g	Tochimoto Tenkaido Co Ltd., Osaka, Japan
Atractylodis Rhizoma	3.0 g	Tochimoto Tenkaido Co Ltd., Osaka, Japan
Poria	3.0 g	Tochimoto Tenkaido Co Ltd., Osaka, Japan
Longan Arillus	3.0 g	Tochimoto Tenkaido Co Ltd., Osaka, Japan
Polygalae Radix	2.0 g	Tochimoto Tenkaido Co Ltd., Osaka, Japan
Zizyphi Fructus	2.0 g	Tochimoto Tenkaido Co Ltd., Osaka, Japan
Angelicae Radix	2.0 g	Tochimoto Tenkaido Co Ltd., Osaka, Japan
Glycyrrhizae Radix	1.0 g	Tochimoto Tenkaido Co Ltd., Osaka, Japan
Saussureae Radix	1.0 g	Tochimoto Tenkaido Co Ltd., Osaka, Japan
Zingiberis Rhizoma	0.375 g	Tochimoto Tenkaido Co Ltd., Osaka, Japan

**Fig. 1:**
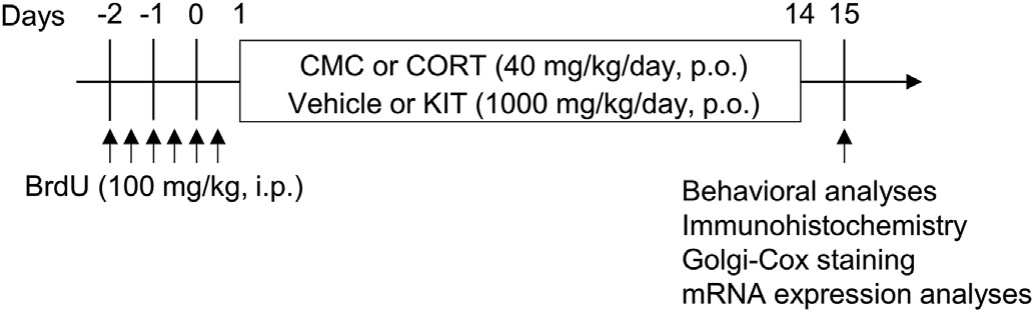
Experimental schedule. Mice were administered CORT (40 mg/kg, s.c.) and/or KIT (1000 mg/kg, p.o.) daily for 14 consecutive days (days 1–14). For analysis of cell survival and the number of new-born immature neurons, BrdU (100 mg/kg/once, i.p.) was administered twice a day for 3 consecutive days before treatment with CORT (from day −2 to 0). The forced swim test, a probe test in the Barnes maze, spontaneous locomotor activity analysis, immunohistochemistry, Golgi-Cox staining and mRNA expression analysis were performed 24 h after the last treatment (day 15).

CORT was purchased from Sigma-Aldrich (St. Louis, MO, USA). Bromodeoxyuridine (BrdU) was purchased from Nacalai Tesque Inc. (Kyoto, Japan). CORT was suspended in 0.5% w/v carboxymethylcellulose (CMC) and KIT was dissolved in distilled water. All drugs were administered at a fixed volume of 10 ml/kg body weight. Mice were randomly assigned to groups treated with CMC/water, CORT/water and CORT/KIT. CMC or CORT (40 mg/kg) were administered subcutaneously and water or KIT (1000 mg/kg) was given orally. These treatments were given daily for 14 consecutive days (days 1–14). BrdU (100 mg/kg/once, i.p.) was administered twice a day for 3 consecutive days before treatment with CORT (from day −2 to 0). The forced swim test, a probe test using the Barnes maze, spontaneous locomotor activity analysis, immunohistochemistry, Golgi-Cox staining and mRNA expression analysis were performed 24 h after the last treatment (day 15). Training sessions in the Barnes maze test were performed in the last 5 days of the treatment period (days 10–14) at 3.5 h before each treatment. The experimental schedule is shown in Fig. 1.

### 2.3 Forced swim test

The forced swim test was carried out as previously described with minor modification.^19^ Briefly, mice were individually placed in a polymethylpentene beaker (height 27 cm, diameter 18 cm) containing 25 ± 1 °C water of depth 13 cm. The performance of mice for 6 min of swimming was videotaped. The total duration of immobility (no movement of the paws and only minimal movement to keep the head above water) was measured in the final 4 min of the 6-min session by an observer blinded to the treatment conditions.

### 2.4 Barnes maze test

The Barnes maze test was carried out as previously described with minor modification.^20^ Briefly, training and probe test sessions were completed on 6 consecutive days: 5 days for training and the last day for the probe test. On the first training day, the mouse was placed directly in the escape box under one of the holes for 180 s to allow it to habituate to the escape box, and this habituation step was repeated three times. After the habituation step, the mouse was placed at the center of the platform and covered with a 500 ml beaker. After 30 s, the mouse was moved to the hole that led to the escape box by dragging the beaker onto the hole and allowed to stay in the escape box for 180 s as pre-training. After this pre-training, the training session was started. The mouse was placed into the white start box (10 × 10 × 10 cm^3^) at the center of the platform. After 30 s, the trial began by removing the start box to allow the mouse to freely explore the platform. If the mouse did not enter the escape box within 300 s in the training session, it was gently moved into the escape box using the beaker. After the mouse had entered the escape box, it was allowed to stay there for 60 s. The training trials were performed three times per day (a total of 15 trials in 5 days) separated by about 90 min. The platform was cleaned with 0.012% sodium hypochlorite before each trial. In the probe test, the escape box was removed from the maze and the mouse was allowed to explore freely for 180 s. The training and probe test trials were videotaped, and the latency to enter the escape box in the training trials and the time spent on each hole in the probe test were measured by an observer blinded to the treatment conditions.

### 2.5 Spontaneous locomotor activity analysis

Each mouse was placed individually in a novel cage (30 × 30 × 30 cm^3^) and locomotor activity was measured for 30 min using ANY-maze video tracking software (Stoelting Co., Wood Dale, IL, USA).

### 2.6 Immunohistochemistry

Immunohistochemistry was performed as previously described.^4^ Briefly, mice were deeply anesthetized with pentobarbital and perfused transcardially with saline, followed by a solution of 4% paraformaldehyde. The brain was fixed with 4% paraformaldehyde over 2 days. Serial 50-μm coronal sections were cut using a microslicer DTK-1000 (Dosaka EM Co., Kyoto, Japan). Six sections per mouse was collected every sixth section between the stereotaxic coordinates −1.4 and −3.2 mm from the bregma, which includes most of the dorsal hippocampus and the ventral hippocampus.

For doublecortin (DCX) staining, free-floating sections were incubated for 1 h with 1% bovine serum albumin (BSA) in PBS containing 0.3% Triton X-100 (PBS-T). After blocking, sections were incubated overnight at 4 °C with anti-DCX goat polyclonal primary antibody solution (1:100 dilution; Santa Cruz Biotechnology, Santa Cruz, CA, USA). After rinsing in PBS, sections were incubated for 1 h at room temperature (RT) with biotinylated anti-goat IgG (1:200 dilution; Vector Laboratories, Burlingame, CA, USA), followed by incubation with Vectastain ABC kit (Vector Laboratories) for 1 h at RT. DCX-positive cells were visualized by incubating sections with Vector SG Peroxidase (HRP) Substrate Kit (Vector Laboratories). For BrdU staining, free-floating sections were incubated in 50% formamide/1 × saline sodium citrate (SSC) for 2 h at 65 °C, followed by a rinse in 2 × SSC. Sections were then incubated in 2 N HCl for 30min at 37 °C (to denature double-stranded DNA) and rinsed in 0.1 M borate buffer (pH 8.5). After blocking for 1 h with 1% BSA in PBS-T, sections were incubated overnight at 4 °C with anti-BrdU rat polyclonal primary antibody (1:100 dilution; Bio-Rad Laboratories, Hercules, CA, USA). After rinsing in PBS, sections were incubated for 1 h at RT with biotinylated anti-rat IgG (1:200 dilution; Vector Laboratories), followed by incubation with Vectastain ABC kit (Vector Laboratories) for 1 h at RT. BrdU-positive cells were visualized by incubating sections with 3,3′-diaminobenzidine (Sigma-Aldrich). The numbers of all cells with brown-stained (BrdU-labeled) nuclei surrounded by blue-gray-stained cell body (DCX-positive) in the dentate gyrus (DG) were counted manually as BrdU-labeled and DCX-immunopositive cells under bright-field illumination at 200 × and 400 × magnification using a microscope with 20 × and 40 × objective lenses and a CCD camera (BX53 with DP73, Olympus Corp., Tokyo, Japan) by an observer blinded to the treatment conditions. The total numbers of BrdU-labeled and DCX-immunopositive cells were obtained by multiplying the counted numbers by six.

### 2.7 Golgi-Cox staining

Golgi-Cox staining was performed using a sliceGolgi Kit (Bioenno Tech, Santa Ana, CA, USA) as previously described.^4^ Briefly, mice were deeply anesthetized and perfused transcardially with saline, followed by the fixative solution. Brains were dissected and postfixed in the fixative solution for 48 h at 4 °C. Serial 100-μm coronal sections containing the hippocampus (−1.4 to −2.4 mm from the bregma) or nucleus accumbens (+1.1 to +0.9 mm from the bregma) were cut using a microslicer DTK-1000 (Dosaka EM Co.). Free-floating sections were incubated in Impregnation Solution for 7 days in the dark. After impregnation, staining and post-staining were performed. Z-stack images were collected and then projected into a single image using the 100 × oil immersion lens of the microscope with a CCD camera (BX53 with DP73, Olympus) and imaging software (cellSens, Olympus). The numbers of spines in secondary dendrites of granule cells in the DG, of medium spiny neurons in the nucleus accumbens, and of pyramidal neurons in CA1 and CA3 were counted. The three neurons were analyzed per section and three distal dendrites to the cell soma were imaged per neuron. The number of spines was averaged from three sections (27 dendrites) per mouse.

### 2.8 Total RNA isolation, reverse transcription and quantitative real-time RT-PCR

Total RNA was isolated from the hippocampus or nucleus accumbens with TRIzol reagent (Thermo Fisher Scientific, Waltham, MA, USA) and used (1 μg) for reverse transcription with ReverTra Ace (Toyobo Co., Osaka, Japan). Quantitative real-time PCR was performed with Thunderbird qPCR Mix (Toyobo) and the primers shown in Table 2, using a Thermal Cycler Dice Real Time System Single (Takara Bio, Shiga, Japan). Changes in gene expression were calculated relative to the endogenous β-actin standard.

**Table 2:** List of primer sequences used in quantitative real-time PCR.

mRNA	Forward primer sequence	Reverse primer sequence
BDNF	CATGAGACCGGGCAAGTC	CCTTGGGAGGAATGTGTGAT
GDNF	GGATGGGATTCGGGCCACT	AGCCACGACATCCCATAACTTC
NGF	TTCTATACTGGCCGCAGTGA	TGTACGGTTCTGCCTGTACG
VEGF	GAGGATGTCCTCACTCGGATG	GTCGTGTTTCTGGAAGTGAGCAA
IGF1	GTGTGGACCGAGGGGCTTTT	GCTTCAGTGGGGCACAGTAC
FGF2	CCAACCGGTACCTTGCTATG	TATGGCCTTCTGTCCAGGTC
NT-3	CCGGTGGTAGCCAATAGAACC	GCTGAGGACTTGTCGGTCAC
β-actin	ACCCACACTGTGCCCATCTA	GCCACAGGATTCCATACCCA

### 2.9 Statistical analysis

**Fig. 2:**
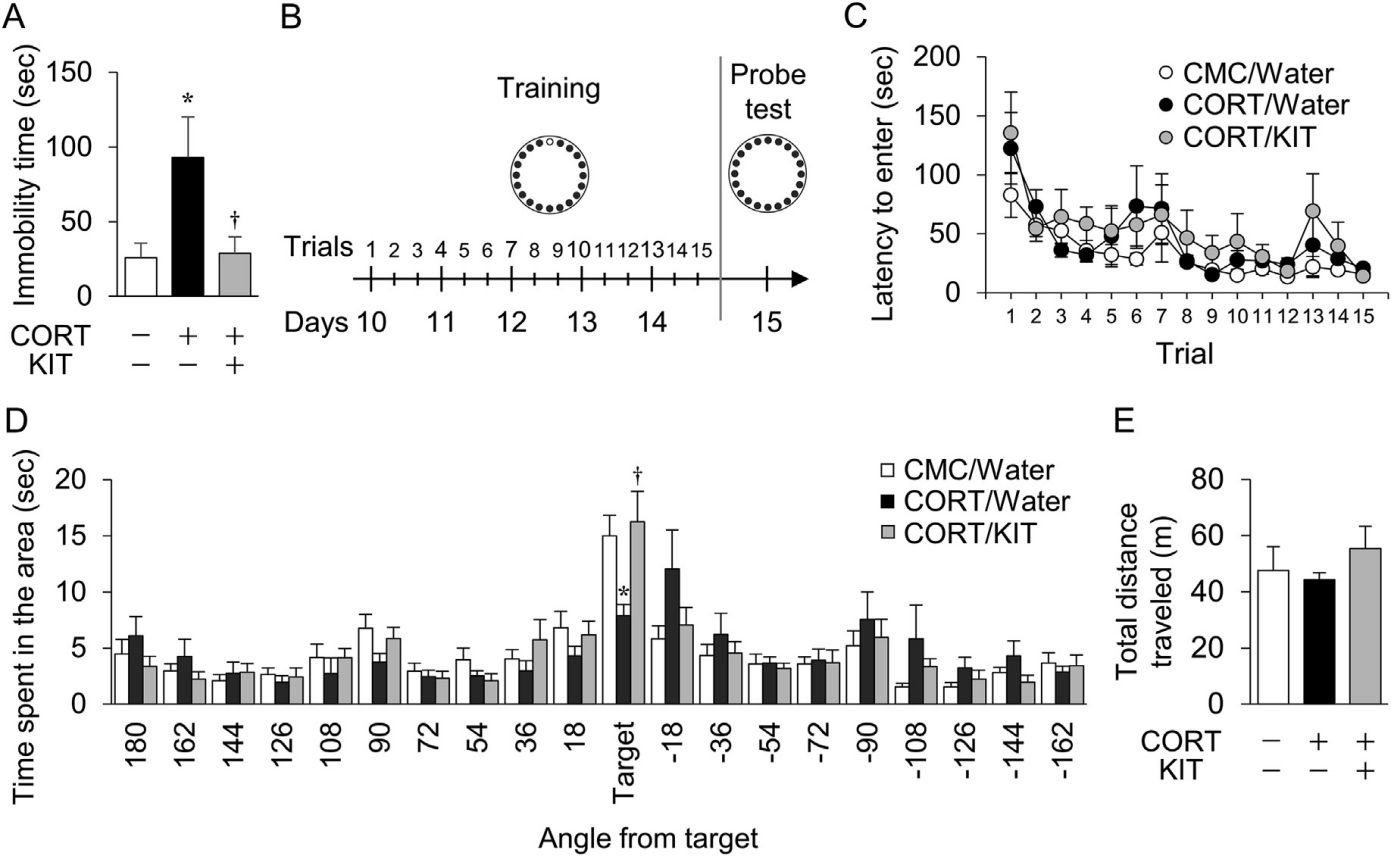
Effects of KIT on depression-like behavior and spatial memory impairment in CORT-treated mice. (A) Immobility time in the forced swim test. (B) Experimental schedule for the Barnes maze test. Small black circles represent closed holes, and a small white circle indicates an open hole (the target hole to the escape box). Training trials were performed three times per day (total of 15 trials for 5 days). In the probe test, the target hole was closed. (C) Latency to enter the escape box during the training session. The mouse was allowed to explore freely for 300 s, and time spent in each hole area was measured. (D) Time spent in the target hole area during the probe test session. The mouse was allowed to explore freely for 180 s, and time spent in the target hole area was measured. (E) Spontaneous locomotor activity. Total distance traveled for 60 min in a novel cage was measured. Values are expressed as mean ± SEM of 12–14 mice for the forced swim test, 11–12 mice for the Barnes maze test, and 5 mice for analysis of spontaneous locomotor activity. ∗*P* < 0.05 vs. CMC/water-treated mice. ^†^*P* < 0.05 vs. CORT/water-treated mice.

**Fig. 3:**
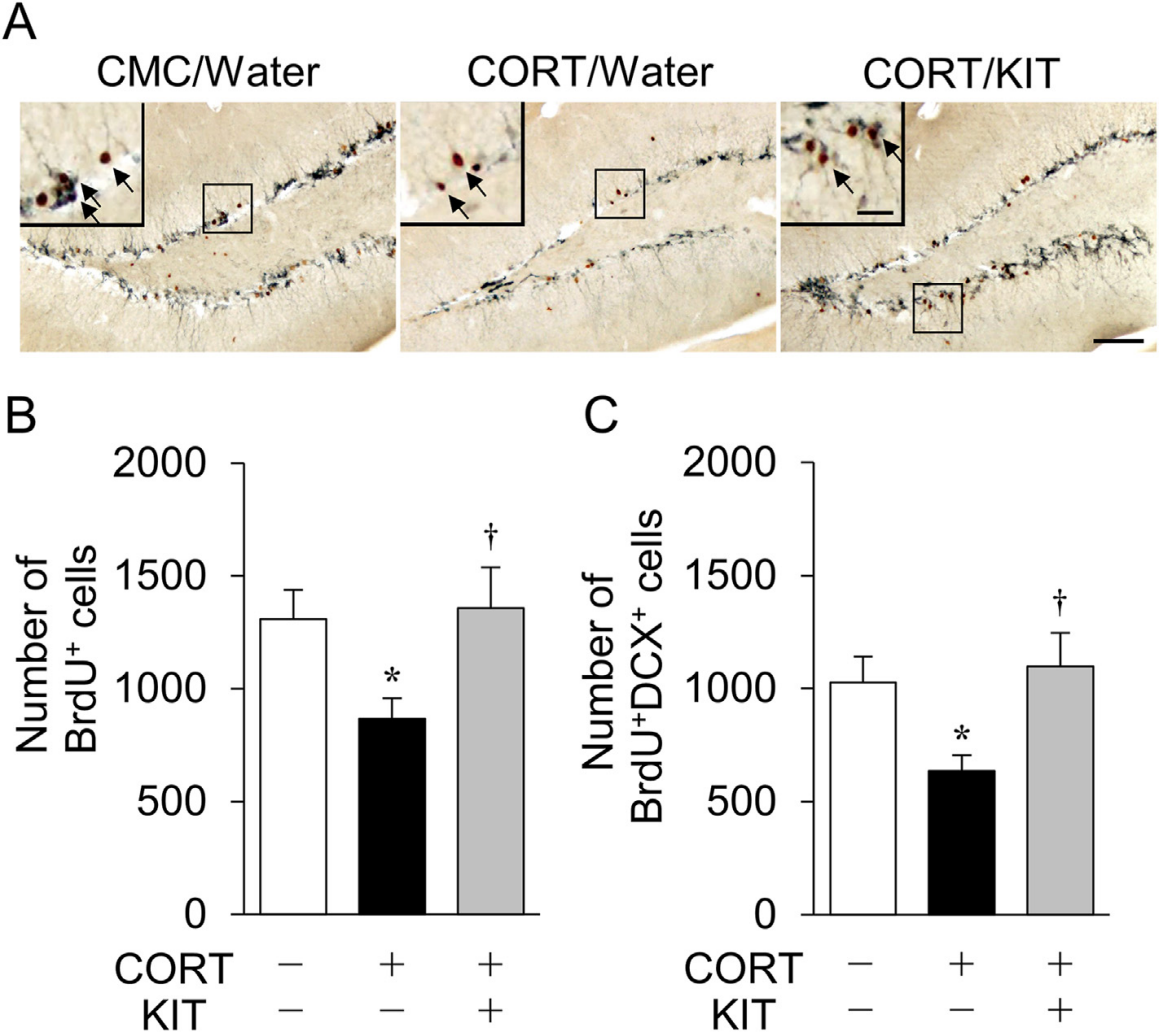
Effects of KIT on CORT-induced reduction of cell survival and the number of new-born immature neurons in the hippocampus. (A) Representative photomicrographs showing DCX (blue-gray)- and BrdU (brown)-immunopositive cells in the dentate gyrus of the hippocampus. Arrows indicate BrdU/DCX double-immunopositive cells. Scale bar: 100 μm. Insert scale bar: 30 μm. (B, C) Number of BrdU-immunopositive cells (B) and BrdU/DCX double-immunopositive cells (C). Values are expressed as mean ± SEM of 10 mice. ∗*P* < 0.05, CMC/water-treated mice. ^†^*P* < 0.05 vs. CORT/water-treated mice.

**Fig. 4:**
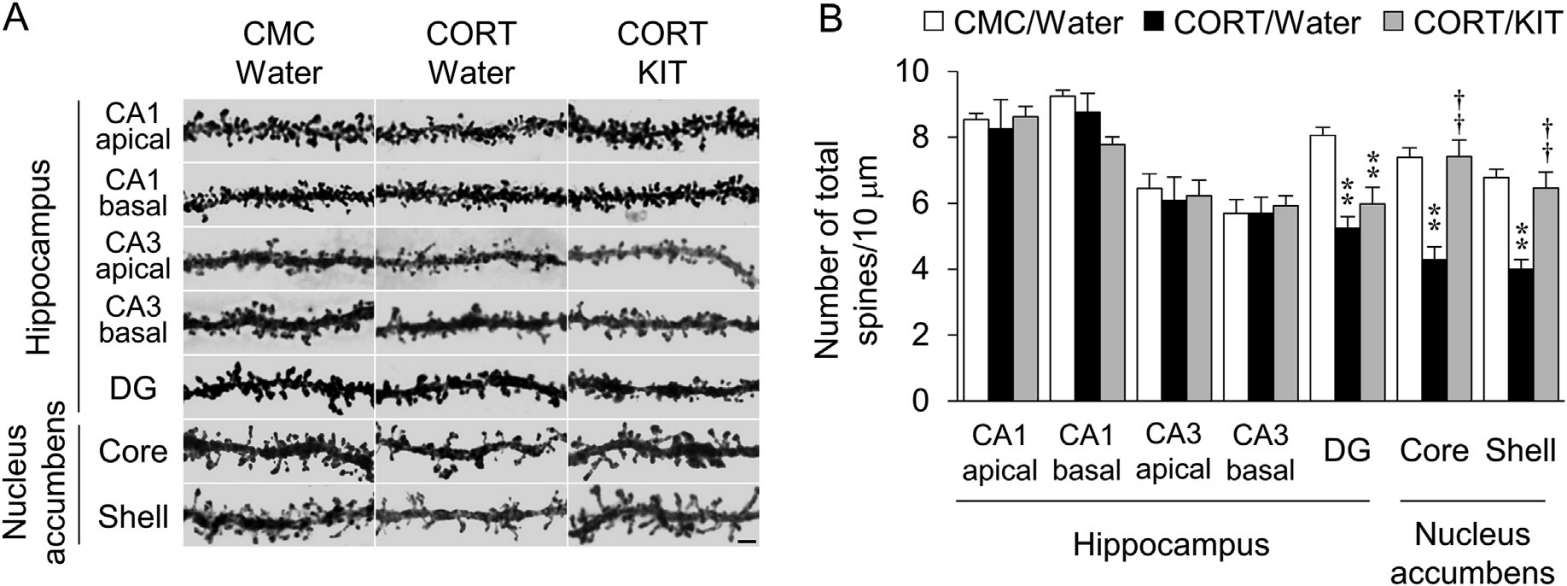
Effects of KIT on CORT-induced decreases in the number of dendritic spines. (A) Representative photomicrographs showing dendritic spines. Scale bar: 1 μm. (B) Number of total spines in the hippocampal CA1, CA3 and DG subregions and in the nucleus accumbens core and shell. Values are expressed as mean ± SEM of 4 mice. ∗∗*P* < 0.01 vs. CMC/water-treated mice. ^††^*P* < 0.01 vs. CORT/water-treated mice.

**Fig. 5:**
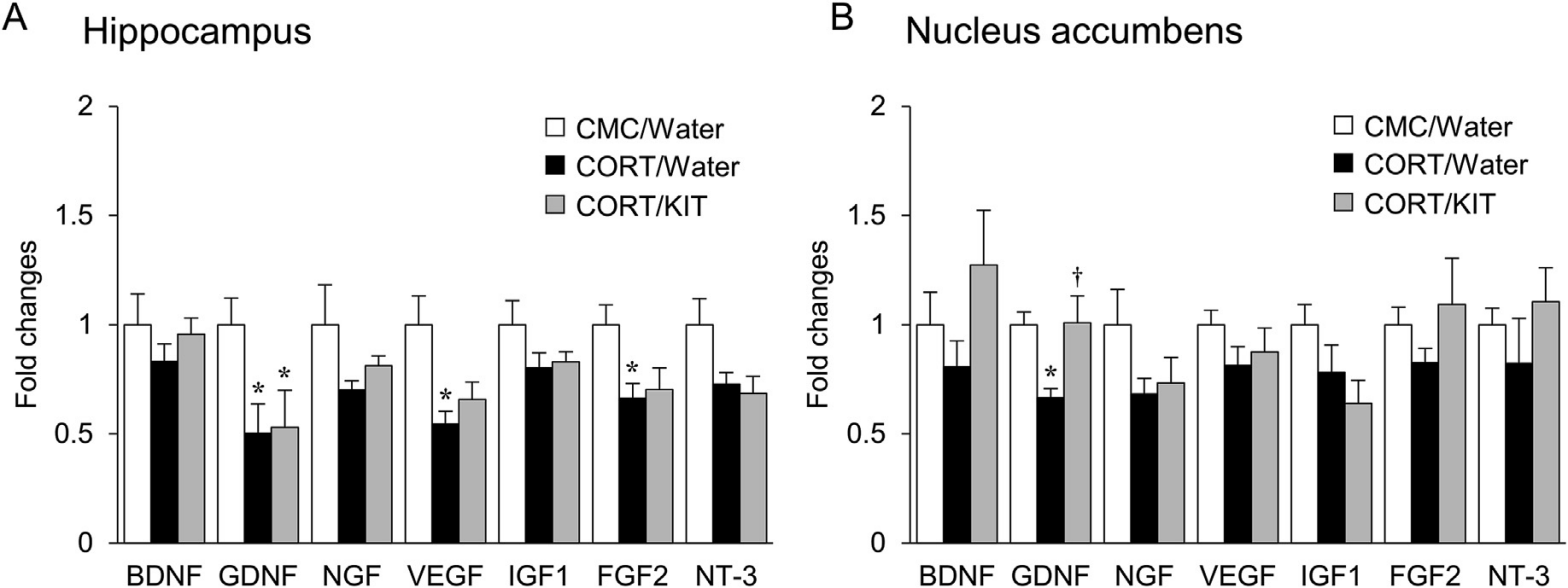
Effects of KIT on CORT-induced decreases in mRNA levels for neurotrophic factors (BDNF, GDNF, NGF, VEGF, IGF1, FGF2 and NT-3) in the hippocampus (A) and nucleus accumbens (B). Values are expressed as mean ± SEM of 8–9 mice. ∗*P* < 0.05 vs. CMC/water-treated mice. ^†^*P* < 0.05 vs. CORT/water-treated mice.

All data are expressed as the mean ± standard error of the mean (SEM). Data in Fig. 2, Fig. 3, Fig. 4, Fig. 5B were analyzed by one-way ANOVA, followed by a Tukey-Kramer *post-hoc* test. Data in Fig. 2C were analyzed by two-way ANOVA for treatment as the intersubject factor and repeated measures with time as the intrasubject factor. All analyses were performed using Statview 5.0J for Apple Macintosh (SAS Institute, Cary, NC, USA). A value of *p* < 0.05 was considered to be significant. The summary statistics of experiments peformed in the study are shown in Supplementary Table S1.

## 3 Results

### 3.1 Effects of KIT on CORT-induced depression-like behavior and spatial memory impairment

First, to investigate the effects of KIT on stress-induced depressed states and spatial memory impairment, the forced swim test and the Barnes maze test were performed. In the forced swim test, the immobility time increased in CORT/water-treated mice compared with CMC/water- or CORT/KIT-treated mice (Fig. 2A). In the Barnes maze test, there were no significant differences among the three groups in latency to enter the escape box in training sessions (Fig. 2B). In contrast, less time was spent in the target area by CORT/water-treated mice compared with CMC/water- or CORT/KIT-treated mice in the probe test session (Fig. 2C). There were no significant differences in spontaneous locomotor activity among the three groups (Fig. 2D).

### 3.2 Effects of KIT on CORT-induced reduction in cell survival and the number of new-born immature neurons in the hippocampus

Next, to investigate the effects of KIT on reduction of cell survival and the number of new-born immature neurons in the hippocampus of CORT-treated mice, we performed BrdU labeling and evaluated newborn neuron development using BrdU and DCX double staining (Fig. 3A). As previously reported,^4^ there were significantly fewer surviving BrdU-positive cells (Fig. 3B) and BrdU/DCX-double positive newborn immature neurons (Fig. 3C) in CORT/water-treated mice compared with CMC/water-treated mice. In contrast, there was no reduction of BrdU-positive or BrdU/DCX-double positive cells in CORT/KIT-treated mice (Fig. 3B and C).

### 3.3 Effects of KIT on CORT-induced decreases in dendritic spine density in the hippocampus and nucleus accumbens

We also examined the effects of KIT on the CORT-induced decrease in dendritic spine density (Fig. 4A). As previously reported,^4^ dendritic spine density was reduced in the DG of the hippocampus and in the nucleus accumbens in CORT/water-treated mice compared with CMC/water-treated mice. In contrast, a decrease in dendritic spine density in the nucleus accumbens was not observed in CORT/KIT-treated mice (Fig. 4B).

### 3.4 Effects of KIT on CORT-induced decreases in mRNA levels for neurotrophic factors in the hippocampus and nucleus accumbens

Effects of KIT on CORT-induced decreases in mRNA levels for neurotrophic factors were also examined. As previously reported,^4^ decreased mRNA levels for GDNF, vascular endothelial growth factor (VEGF) and fibroblast growth factor 2 (FGF2) were found in the hippocampus of CORT/water-treated mice (Fig. 5A), and GDNF mRNA was also lower in the nucleus accumbens of these mice (Fig. 5B). KIT significantly reversed the CORT-induced decreases in these mRNA levels in the nucleus accumbens, but not in the hippocampus (Fig. 5A and B).

## 4 Discussion

In this study, behavioral analyses revealed that KIT suppressed CORT-induced depression-like behavior and spatial memory impairment, as indicated by increased immobility in the forced swim test and decreased time spent in the target area in the probe test of the Barnes maze test, respectively. These results reproduce the effects of KIT on a depressed state and dementia in clinical practice, and support the usefulness of this mouse model in investigations of the effects of KIT on stress-induced brain dysfunctions.

Chronic CORT treatment reduced cell survival and number of new-born immature neurons in the hippocampus, as indicated by decreased numbers of BrdU-positive and BrdU/DCX double-positive cells, respectively. Reduction of neurogenesis including reduced cell survival and number of new-born immature neurons is thought to be one of the molecular mechanisms underlying stress-induced depressive symptoms^7^ and spatial reference memory impairments.^8^ In this study, KIT prevented CORT-induced reduction in cell survival and the number of new-born immature neurons, which suggests that protection of hippocampal new-born immature neurons from the actions of glucocorticoids may underlie the ameliorative effects of KIT on stress-induced brain dysfunctions. Since chronic administration of CORT is known to reduce the volume of the hippocampus,^21^ it is also possible that the decrease in the number of newborn immature neurons observed in this study is due to the reduced hippocampal volume. Whether the effects of KIT are mediated by the prevention of CORT-induced reduction in hippocampal volume require further investigation.

KIT also prevented CORT-induced decreases in dendritic spine density in the nucleus accumbens. The contribution of this spine density to development of depressive symptoms is controversial. However, since the nucleus accumbens plays an important role in processing reward stimuli, dysfunctions of the nucleus accumbens are thought to be linked to depression accompanied by diminished interest or pleasure.^22^ Furthermore, several studies have shown abnormalities of dendritic spine density and maturation in the nucleus accumbens in animal models of depression.^10^ Given these reports, effects on dendritic spine density and maturation in the CA1 and nucleus accumbens may be involved in the preventive effects of KIT on stress-induced cognitive impairments and depressive symptoms.

There is accumulating evidence for a correlation between depressive symptoms and GDNF levels. Clinical studies have reported lower serum GDNF in patients with major depressive disorder and increases in serum GDNF by antidepressant treatment.^23^ Stressed mice have lower GDNF in the nucleus accumbens, and this GDNF level is increased by antidepressant treatment.^24^ In this study, KIT prevented the CORT-induced decrease in accumbal GDNF, which suggests that KIT may improve a stress-induced depressed state by maintaining normal GDNF levels in the nucleus accumbens.

We have previously shown that an aqueous extract of Polygalae Radix, a component herb of KIT, suppresses depression-like behavior, abnormalities in accumbal dendritic spines and decreases in accumbal GDNF, but does not affect spatial memory impairment or reduced hippocampal cell survival and number of hippocampal new-born immature neurons, in CORT-treated mice.^4^ These results suggest that Polygalae Radix may play a part in the effects of KIT on CORT-induced depression-like behavior, abnormalities in accumbal dendritic spines and decreases in accumbal GDNF. In contrast, the preventive effects of KIT on CORT-induced memory impairment or reduced hippocampal cell survival and number of hippocampal new-born immature neurons seem to be caused by another component. Lim et al. showed that Ginseng Radix, a component herb of KIT, increases cell proliferation in the dentate gyrus of diabetes model rats,^25^ and Jiang et al. found that ginsenoside Rg1, a constituent of Ginseng Radix, ameliorates chronic mild stress-induced depression-like behavior and reduced hippocampal neurogenesis.^26^ Several studies have shown that extracts or constituents of Zizyphi Semen, a component herb of KIT, ameliorates cognitive and memory impairment and reduced hippocampal neurogenesis in several animal models.^27^, ^28^, ^29^, ^30^, ^31^ These component herbs in KIT may act synergistically with Polygalae Radix.

In conclusion, this study suggests that KIT may ameliorate a stress-induced depressed state and memory impairment via prevention of adverse effects of stress on hippocampal cell survival, the number of new-born hippocampal immature neurons, accumbal dendritic spines and accumbal GDNF levels.

## Declaration of competing interest

The authors declare no conflicts of interest.
