# Supplementary material for: Kihito prevents corticosterone-induced brain dysfunctions in mice
Source: J Tradit Complement Med. 2021 May 15;11(6):513–9. doi: 10.1016/j.jtcme.2021.05.002 (PMC8572719; doi:10.1016/j.jtcme.2021.05.002)
Supplement: Multimedia component 2 [file mmc2.docx]

**Liquid chromatography-mass spectrometry (LC-MS) analyses**

The chemical profiling of kihito (KIT) extract using LC-MS technique was performed with a Shimadzu LC-IT-TOF mass spectrometer equipped with an ESI interface (Shimadzu, Kyoto, Japan). The ESI parameters were as follows: source voltage, +4.5 kV (positive ion mode) or –3.5 kV (negative ion mode); capillary temperature, 200°C; nebulizer gas, 1.5 l/min. The mass spectrometer was operated in positive and negative ion mode scanning from m/z 100 to 2000. A Waters Atlantis T3 column (2.1 mm i.d. × 150 mm; Nihon Waters K.K., Tokyo, Japan) was used and the column temperature was maintained at 40°C. The mobile phase was a binary eluent of A) water containing 0.1% (v/v) formic acid, and B) CH_3_CN containing 0.1% (v/v) formic acid, under the following gradient conditions: 0-30 min linear gradient from 10% to 100% B, then 30-40 min isocratic at 100% B. The flow rate was 0.2 ml/min. Mass spectrometry data obtained from the extract have been stored together with pharmacological information on the extracts in the Wakan-Yaku DataBase system (http://dentomed.toyama-wakan.net/en/information_on_experimental_kampo_extracts/kamiuntanto%20extract-2017-KM/EXP006002, Institute of Natural Medicine, University of Toyama).

**Supplementary table notes**

**Supplementary table.** Summary of statistics in the study.
